# Lack of Association between Membrane-Type 1 Matrix Metalloproteinase Expression and Clinically Relevant Molecular or Morphologic Tumor Characteristics at the Leading Edge of Invasive Colorectal Carcinoma

**DOI:** 10.1155/2015/185404

**Published:** 2015-05-27

**Authors:** Annette Arndt, Klaus Kraft, Eva Wardelmann, Konrad Steinestel

**Affiliations:** ^1^Institute of Pathology and Molecular Pathology, Bundeswehrkrankenhaus Ulm, 89081 Ulm, Germany; ^2^Gerhard-Domagk-Institute of Pathology, University Hospital Münster, 48149 Münster, Germany

## Abstract

Colorectal cancer (CRC) is one of the leading causes of death from cancer in the western world, but tumor biology and clinical course show great interindividual variation. Molecular and morphologic tumor characteristics, such as *KRAS/BRAF* mutation status, mismatch repair (MMR) protein expression, tumor growth pattern, and tumor cell budding, have been shown to be of key therapeutic and/or prognostic relevance in CRC. Membrane-type 1 matrix metalloproteinase (MT1-MMP) is a membrane-anchored zinc-binding endopeptidase that is expressed at the leading edge of various invasive carcinomas and promotes tumor cell invasion through degradation of the extracellular matrix. The aim of this study was to investigate possible associations between MT1-MMP expression and molecular tumor characteristics as well as morphologic features of tumor aggressiveness in a consecutive series of 79 CRC tissue samples. However, although MT1-MMP was expressed in 41/79 samples (52%), there was no significant association between MT1-MMP expression and *KRAS/BRAF* mutation status, MMR protein expression, presence of lymphovascular invasion, tumor growth pattern, tumor-infiltrating lymphocytes, or tumor cell budding in our sample cohort (*P* > 0.05). Thus, we conclude that although MT1-MMP may play a role in CRC invasion, it is not of key relevance to the current models of CRC invasion and aggressiveness.

## 1. Introduction

Worldwide, colorectal carcinoma (CRC) is the third most common cancer in men and the second most common cancer in women [1]. Invasion and metastatic dissemination of tumor cells via blood and/or lymph vessels are key determinants of patient prognosis [2, 3]. However, although tumor staging according to TNM/UICC provides relevant prognostic information in general, the individual outcomes between otherwise comparable patients vary to a great extent; this is true in particular for UICC stage II and III carcinomas [4]. This limitation has motivated researchers to identify additional risk factors and biologic subgroups that might help to improve patient stratification and the resulting therapy decisions. Multiple histomorphologic and molecular tumor characteristics have been shown to be significantly associated with CRC aggressiveness: patients with microsatellite-instable (MSI) tumors with a resulting loss of DNA mismatch repair (MMR) protein expression frequently show high numbers of tumor-infiltrating lymphocytes (TILs), and while these features are associated with a better prognosis, a diffusely infiltrating tumor growth pattern correlates with poor outcome in rectal cancer [5–7]. These findings have since then been confirmed by multiple independent studies, underscoring the prognostic value of these characteristics in CRC [8, 9].

Tumor cell budding, defined as invasion by single tumor cells or small clusters of cells at the leading edge, is an independent adverse prognostic factor in CRC and predicts response to antiepidermal growth factor receptor (EGFR) therapy [8, 10]. This also applies to activating mutations in* RAS* oncogenes (detectable in up to 45% of CRCs) and is the reason why* NRAS/KRAS* mutational testing is nowadays routinely performed in patients with metastatic CRC (mCRC) [11, 12]. The presence of an activating* BRAF* mutation, on the other hand, does not seem to predict anti-EGFR therapy response but is significantly associated with poor survival especially in the microsatellite-stable (MSS) setting [13, 14].

Given the predictive and/or prognostic value of the above-mentioned variables, we have analyzed possible correlations between morphologic and molecular markers in CRC to identify a possible biological pattern behind more aggressive tumor behavior in a previous study [15]. However, in that study, while confirming the association between expanding tumor growth, tumor-infiltrating lymphocytes, and loss of MMR protein expression, there was no such association between the presence of* KRAS*/*BRAF* mutations and a certain growth pattern or budding intensity in CRC [15].

Membrane-type 1 matrix metalloproteinase (MT1-MMP) is a membrane-anchored zinc endopeptidase and a key enzyme in degradation of the pericellular extracellular matrix (ECM) [16]. It is delivered to the leading edge of invading cancer cells and, besides its ability to digest ECM components such as fibronectin, vitronectin, and collagens I–III, is capable of inducing functional conversion of target molecules, such as matrix metalloproteinase 2, CD44, integrin, and laminin. In accordance to its central role in ECM degradation, it has been shown that MT1-MMP expression is essential for invasion of fibrosarcoma, gastric and breast cancer, and hepatocellular carcinoma cells [17–19]. In prostate cancer, overexpression of MT1-MMP induces epithelial-mesenchymal transition, a process where cells lose epithelial and gain fibroblast-like characteristics in support of a proinvasive phenotype [20, 21].

In colon cancer, it has been shown that MT1-MMP is frequently upregulated downstream of the Wnt pathway signaling as a target gene for *β*-catenin; MT-MMP1-mediated cleavage of laminin 5 supports a migratory phenotype in CRC cells [22, 23]. Furthermore,* MT1-MMP* gene expression, although unrelated to any other established clinic-pathologic feature, has been reported to be an independent prognostic factor for overall survival in CRC [24]. However, to the best of our knowledge, a possible association between MT1-MMP protein expression and molecular (*KRAS*/*BRAF* mutation status) and morphologic tumor characteristics (invasion pattern/budding) has so far not been investigated. Therefore, the aim of this study was to elucidate a possible link between clinically relevant molecular or morphologic tumor subtypes and MT1-MMP expression at the leading edge of invasive CRC.

## 2. Materials and Methods

### 2.1. Ethics Statement

The current study is part of a project that received institutional review board approval from the ethics committee of the University of Ulm (Number 162/13). Other results obtained from this cohort of CRC patients have been previously published [15, 25].

### 2.2. Tissue Samples and Morphologic Classification

Seventy-nine consecutive cases of invasive adenocarcinoma of the colon and rectum were included in the study as previously described [15]. In short, clinical data included patient age, tumor localization, and the presence of lymph node or organ metastasis. For each case, one representative paraffin-embedded tissue block containing the invasive margin of the tumor was selected and multiple 4 *μ*m sections where cut for hematoxylin/eosin (HE) and immunohistochemical (IHC) stainings, respectively. Peritumoral lymphocytic (PTL) infiltrate, configuration of the invasion margin (expanding/infiltrating, following the criteria by Jass et al. [6, 8]), tumor cell budding [8], tumor grade (well/moderately/poorly differentiated), and lymphovascular/venous invasion were assessed by one of the authors (KS) under supervision of an experienced pathologist (KK). For assessment of PTL infiltrate, we defined the following key criteria from the list of criteria that has been originally proposed by Jass et al. (1996): (1) presentation as a loose connective tissue lamina or cap; (2) a “lichenoid” type arrangement of inflammatory cells; (3) macrophages, eosinophils, and plasma cells that may be interposed between lymphocytes and glands [6]. When the inflammatory infiltrate at the invasive margin fulfilled two of these three criteria, the case was valued as “PTL positive.” Tumor cell budding was defined according to the criteria by Mitrovic et al. (number of isolated single tumor cells/clusters of fewer than five cells in a 20x objective field, referred to as “intensity” of tumor cell budding) [26]. In “borderline” cases (5–10 definite buds/20x field with possible additional tumor cells), IHC for Pan-Keratin was performed (see below).

### 2.3. DNA Isolation and* KRAS/NRAS/BRAF* Mutation Testing

DNA isolation and pyrosequencing of* KRAS/NRAS/BRAF* hotspot mutations were performed as previously described [15]. In short, sections of the tissue block were cut and transferred into 1.5 mL tubes. DNA extraction was carried out automatically using the Maxwell 16 instrument and the Maxwell 16 FFPE LEV DNA purification kit (both from Promega, Mannheim, Germany) according to the manufacturer's instructions after Proteinase K digestion (conc. 10 mg/mL, 70°C overnight; Promega, Mannheim, Germany). The presence of mutations in codons 12, 13, and 61 of the* KRAS* and* NRAS* genes and in codon 600 of the* BRAF* gene was determined using the Pyromark Q24 pyrosequencing platform and the IVD approved therascreen* KRAS, NRAS,* and* BRAF* pyro kits (Qiagen, Hilden, Germany) according to the respective protocols.

### 2.4. Immunohistochemistry

**Figure 1:**
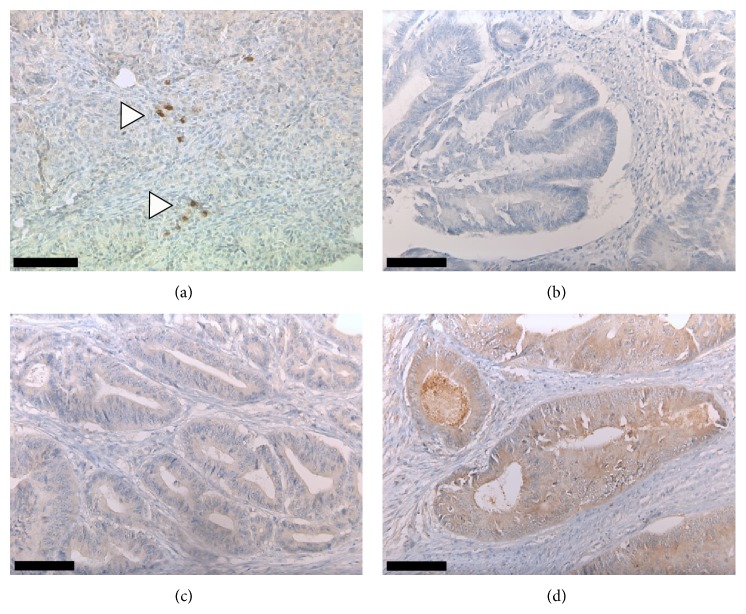
(a and b) Absence of MT1-MMP expression at the leading edge of invasive CRC. Note strong MT1-MMP immunostaining of tumor-associated dendritic cells which serves as internal positive control (*arrowheads*). (c) Weak and (d) strong positivity for MT1-MMP at the leading edge of invasive CRC.* Scale bar* (a)–(d): 100 *μ*m.

Immunohistochemistry was performed on a BenchMark autostainer (Ventana Medical Systems, Tucson, Arizona, USA) according to the manufacturer's protocol. The following monoclonal antibodies were used in the study: MT1-MMP (catalytic domain, clone 114-6G6), 1 : 100, mouse (from Merck Millipore, Darmstadt, Germany); Pan-Keratin (clone AE1/AE3/PCK26), mouse; MLH1 (clone G168–728), mouse; MSH2 (clone G219–1129), mouse; MSH6 (clone 44), mouse; PMS-2 (clone EPR3947), rabbit (prediluted; obtained from Ventana Medical Systems, Tucson, Arizona, USA). For MT1-MMP, cases with weak to strong cytoplasmic and/or membranous immunostaining at the leading edge of the tumor were classified as MT1-MMP positive (Figure 1). Loss of MMR protein expression required lack of nuclear immunostaining for MLH1, MSH2, MSH6, or PMS-2 in tumor cells with retained positivity in nonneoplastic epithelium, stromal and immune cells [27].

### 2.5. Statistical Analysis

Possible associations between intensity of MT1-MMP immunostaining and molecular (*KRAS* codon 12/13 mutation,* BRAF* codon 600 mutation, and MMR deficiency) or morphologic criteria (lymph/blood vessel infiltration, budding intensity, and tumor growth pattern) were evaluated applying Chi-square and Fisher's exact test, respectively. GraphPad Prism 6 software (GraphPad, La Jolla, California, USA) was used for all statistical analyses. A *P* value of <0.05 was regarded as statistically significant.

## 3. Results

### 3.1. Clinicopathologic Characteristics

Tissue samples from 79 patients (median age 75 years, range 25–92 years, IQR 15 years) were analyzed in this study. Clinicopathologic sample characteristics are summarized in Table 1. All UICC stages were included in the sample set; with regard to molecular tumor characteristics,* KRAS* mutations were present in 27/76 (36%) while* BRAF* mutations were present in 9/76 (12%) of samples. 13 of the 40* KRAS*/*BRAF*-wt cases were additionally tested for the presence of* NRAS* mutations, revealing one* NRAS* Q61H mutation. 8/68 tumors (12%) showed loss of MMR protein expression. Morphologic hallmarks of tumor aggressiveness such as presence of lymph and/or blood vessel infiltration or high-grade tumor cell budding were detected in 41/76 (54%) and 19/76 (25%) samples, respectively. 30/77 tumors (39%) displayed an infiltrating growth pattern with ill-defined borders, while tumor-infiltrating lymphocytes were present in 28/77 cases (36%).

### 3.2. MT1-MMP Immunostaining

Positive immunostaining for MT1-MMP at the leading edge was detected in 41/79 CRC specimens (52%, Figure 1 and Table 1). Of these, 38 tumors (93%) showed weak to moderate while 3 tumors (7%) showed strong staining intensity. 38 tumors (48%) were negative for MT1-MMP expression. Strong MT1-MMP immunostaining of tumor-associated dendritic cells has been previously described and served as internal positive control [28]; however, staining intensity of dendritic cells exceeded the intensity of MT1-MMP immunostaining of tumor cells. In MT1-MMP positive tumor cells, immunopositivity was confined to the cytoplasm and to the cell membrane, while nuclei were negative for MT1-MMP (Figure 1).

### 3.3. MT1-MMP Expression and Molecular and Morphologic Tumor Characteristics

MT1-MMP immunostaining did not correlate with histopathological tumor grade or UICC stage (*P* = 1.0 and 0.893, resp.). There was no association between MT1-MMP expression and depth of tumor invasion (T stage) or lymph node involvement (N stage; *P* = 0.633 and 1.0). With regard to molecular tumor characteristics, MT1-MMP expression did neither correlate with the presence of an activating* KRAS* or* BRAF* mutation nor correlate with MMR protein expression at the leading edge of the examined tumors in our sample set (*P* = 0.812, 1.0, and 0.471, resp.). Analysis of a possible correlation between the combined* KRAS*/*BRAF* mutation status and MT1-MMP expression also failed to reach statistical significance (*P* = 0.653). With regard to morphologic hallmarks of tumor aggressiveness, there was no association between MT1-MMP expression at the leading edge of the tumor and the presence of lymphovascular invasion, high-grade tumor cell budding or an infiltrating growth pattern in our sample set (*P* = 0.336, 0.609, and 0.641, resp.). Finally, there was no significant correlation between MT1-MMP expression and the presence of tumor-infiltrating lymphocytes (*P* = 1.0).

## 4. Discussion

Molecular and morphologic characteristics that predict therapy response or aggressive tumor biology are of help in the risk stratification of patients with invasive CRC. Since MT1-MMP is a key enzyme in the degradation of the extracellular matrix and helps in the functional conversion of biologically relevant target molecules, it is of central importance during tumor cell migration and invasion as a prerequisite for metastatic spread. Therefore, the aim of this study was to examine MT1-MMP expression at the leading edge of invasive CRC and to elucidate possible associations with molecular or morphologic tumor characteristics.

The investigated cohort might be regarded as representative in terms of patient and tumor characteristics, and tumors of all UICC stages were included in the study [4]. The proportions of* KRAS*- and* BRAF-*mutant tumors among our sample set (36% and 12%, resp.) also reflect data that has been reported by other authors [12, 29]. The frequency of BRAF mutations was slightly higher in our sample set because one of our initial aims was to evaluate whether the reported aggressiveness of MSS/*BRAF-*mutant tumors is linked to MT1-MMP expression [30]. Loss of MMR protein expression as an indicator for microsatellite instability was observed in 12% of tumors, comparable to literature data [31].

Blood and/or lymph vessel invasion was present in 54% of cases, which is in line with published data ranging from 11 to 89.5% (reviewed in [32]); we found high-grade tumor cell budding in 25% of cases, consistent with data from literature [8]. For both characteristics, however, it should be noted that although their association with an adverse outcome is widely accepted, comparability between studies is significantly hampered by the lack of standardized evaluation (reviewed in [32]). In the present study, we employed the widely used classification for tumor cell budding in CRC that has initially been introduced by Ueno et al. in the slightly modified version as reviewed by Mitrovic et al. [8, 26]. We have previously reported a significant correlation between high-grade tumor cell budding, infiltrating growth pattern and lymph and/or blood vessel infiltration among the cases presented here in another study, underscoring the value of these features as indicators of tumor aggressiveness [15].

Expression of MT1-MMP together with nuclear *β*-catenin and the MT1-MMP substrate laminin-2 chain at the leading edge of invasive CRC has been previously described [23]; additionally, the authors of that study showed *β*-catenin-mediated activation of MT1-MMP transcription in CRC cells* in vitro*, confirming similar results from a previous paper [22]. While MT1-MMP mRNA expression levels correlated with advanced TNM stage, but not vascular invasion in one study, weak expression of MT1-MMP has been reported to be associated with favourable survival in CRC in another study [33, 34]. Interestingly, treatment with the tyrosine kinase inhibitor STI571/Imatinib (Glivec) reduced CRC cell growth, MMP-2 activation, and MT1-MMP expression in another study [35]; accordingly, we have previously shown that Imatinib significantly reduces tumor cell adhesion, ECM degradation, and invasion by CRC cells through inhibition of Abi1 phosphorylation, a key regulator of cytoskeletal dynamics during cell migration [25]. While these findings indicate a central role for MT1-MMP in CRC tumorigenesis and progression, there was no association between expression of the protein and infiltrative tumor growth, tumor cell budding, or lymphovascular invasion in our sample set. Moreover, MT1-MMP protein expression was not associated with more advanced UICC stage. These findings stand in contrast to previously published results that link MT1-MMP expression to depth of tumor invasion and blood vessel infiltration [36]; in the same paper, however, there was no significant association between MT1-MMP expression and lymph vessel infiltration, lymph node, or distant metastasis in CRC, in line with our findings.

Although some reports indicate MT1-MMP upregulation upon activating* KRAS* mutations, there was no significant association between* KRAS* or* BRAF* status and MT1-MMP expression in our sample set [37]. There was also no significant correlation between hallmarks of MSI-H tumors (expanding growth pattern, loss of MMR protein expression, and presence of tumor-infiltrating lymphocytes) and MT1-MMP expression; this finding, on the other hand, is in line with previous reports from other groups [38]. It has to be stated as a clear limitation to our study that not all* KRAS*/*BRAF*-wt samples were also tested for* NRAS* mutations. However, the presence of one* NRAS* Q61H mutation among the 13 tested samples suggests that although the prevalence of RAS isoform mutations might be slightly underrated in our cohort, the expected number of additional* NRAS* mutations would not alter the statistical significance of the findings.

Taken together, although MT1-MMP immunostaining was positive in the majority of examined samples here (52%) and partly contradictory to results previously reported, expression of the protein was not associated with any molecular or morphologic tumor feature in our sample set. Moreover, MT1-MMP immunostaining was stronger in tumor-associated dendritic cells than in the tumor cells. One possible explanation is that although MT1-MMP plays a central role in tumor cell invasion in a variety of malignancies, its mode of action is dependent on the interaction with other members of the metalloproteinase family as well as tissue inhibitors of metalloproteinases (TIMPs); accordingly, hierarchical cluster analysis including IHC sores for MMP-1, MMP-2, MMP-3, MMP-7, MMP-9, MMP-13, MT1-MMP, and MT2-MMP as well as TIMP-1, TIMP-2, and TIMP-3 successfully identified a subgroup of stage III CRCs with poor prognosis in a previous study [39]. This indicates that the individual MMP/TIMP composition at the leading edge of each tumor might create a fine-tuned microenvironment irrespective of (and of higher prognostic relevance than) expression of one MMP or baseline oncogenic mutations, such as* KRAS* or* BRAF*. With that in mind, further studies should focus on the leading edge of tumors and take into account the complete MMP/TIMP network to gain a more detailed insight in the biology of ECM degradation and tumor cell invasion as a prerequisite for CRC metastasis. This knowledge might then allow for the development of specific MMP inhibitors to prevent the gain of a metastatic phenotype in CRC.

## Figures and Tables

**Table 1:** Clinicopathologic sample characteristics and MT1-MMP expression.

Number of patients	79		

m/f	45/34		

Age (yrs, median/range/IQR)	75/25–92/15		

Tumor localization (right/left colon/unknown)	27/48/4		

MT1-MMP expression	MT1-MMP−	MT1-MMP+	*P *

Histopathological grading (*n* = 76)			
Low-grade	15	16	1.0
High-grade	21	24	

UICC stage (*n* = 78)			
I	11	16	0.893^4^
II	10	7	
III	11	14	
IV	4	5	

T stage (*n* = 75)			
T1/2	12	16	0.633
T3/4	24	23	

N stage (*n* = 71)			
N0	20	21	1.0
N1/2	14	16	

*KRAS* mutation status (*n* = 76)			
*KRAS*wt	24	25	0.812
*KRAS*mut^1^	12	15	

*BRAF* mutation status (*n* = 76)			
*BRAF*wt	32	35	1.0
*BRAF*mut^2^	4	5	

MMR protein expression (*n* = 68)			
MMR proteins expressed	32	28	0.471
Loss of MMR protein expression^3^	3	5	

Presence of lymphovascular invasion (*n* = 76)			
L0V0	14	21	0.336
L1/V1	15	13	
L1V1	8	5	

Tumor cell budding (*n* = 76)			
Low-grade	26	31	0.609
High-grade	10	9	

Growth pattern (*n* = 77)			
Expanding	24	23	0.641
infiltrating	13	17	

Tumor-infiltrating lymphocytes (TILs; *n* = 77)			
Absent	24	25	1.0
Present	13	15	

^1^KRAS G12D, G12V, G12C, G12S, and G12R; G13D; Q61K; Q61L; ^2^BRAF V600E; ^3^MLH-1, MSH-2, MSH-6, and PMS-2; ^4^Chi-Square test for trend.
